# Supplementary material for: Reversible displacive transformation in MnTe polymorphic semiconductor
Source: Nat Commun. 2020 Jan 3;11:85. doi: 10.1038/s41467-019-13747-5 (PMC6941995; doi:10.1038/s41467-019-13747-5)
Supplement: Supplementary file 1 — Supplementary Information [file 41467_2019_13747_MOESM1_ESM.pdf]

Supplementary Information

Reversible displacive transformation in MnTe polymorphic semiconductor

Mori et al.

Correspondence to: ysutou@material.tohoku.ac.jp (Y. Sutou)

Table of contents:

Supplementary Figures 1 to 14
Supplementary Tables 1 to 2
Supplementary Note 1. X-ray diffraction measurement for the as-deposited MnTe film without a W capping layer
Supplementary Note 2. Transmission electron microscopy microstructure of MnTe films
Supplementary Note 3. Resistive switching behavior of the MnTe and GST225 devices
Supplementary Note 4. Operation energy of the memory device
Supplementary Note 5. Simulated high-resolution TEM and annular dark-field scanning TEM images analyzed by the multislice method
Supplementary Note 6. Transition temperature from the α-phase to the β′-phase
Supplementary Note 7. Estimation of an expected strain in the MnTe layer of the device
Supplementary Note 8. Transition temperature from the β′-phase to the α-phase
Supplementary Note 9. Thickness dependence of transformation temperature
Supplementary Note 10. Thermal stability of the RESET-operated MnTe device
Supplementary Note 11. Reflectance and transmittance in a wide wavelength range
Supplementary References

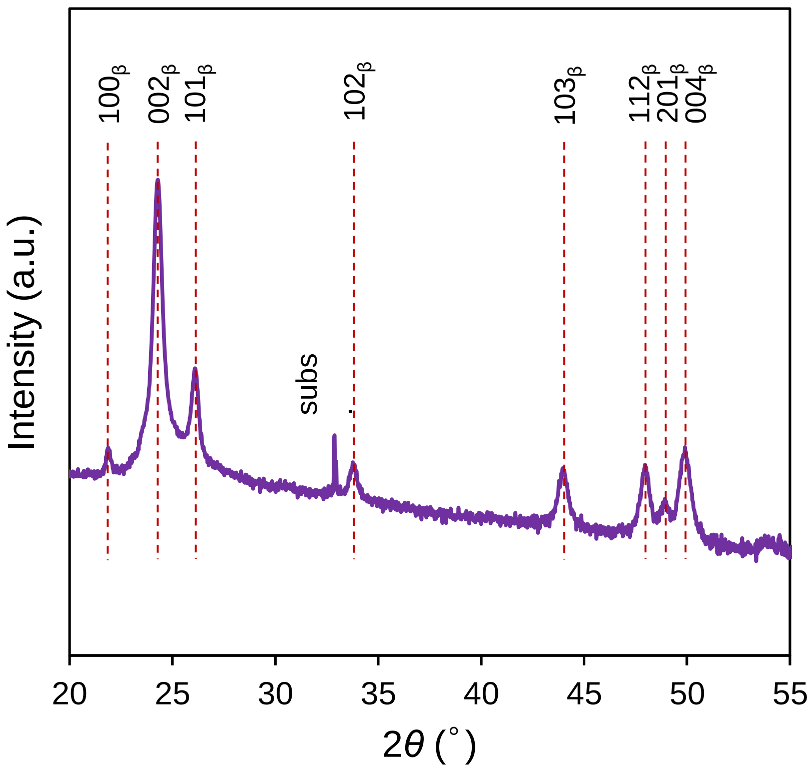

Supplementary Figure 1. X-ray diffraction pattern of the as-deposited MnTe film. The thickness of the film was 1 μm and no W cap layer was deposited on the film. All Bragg reflection peaks indicates the wurtzite-type structure of β-crystalline phase.

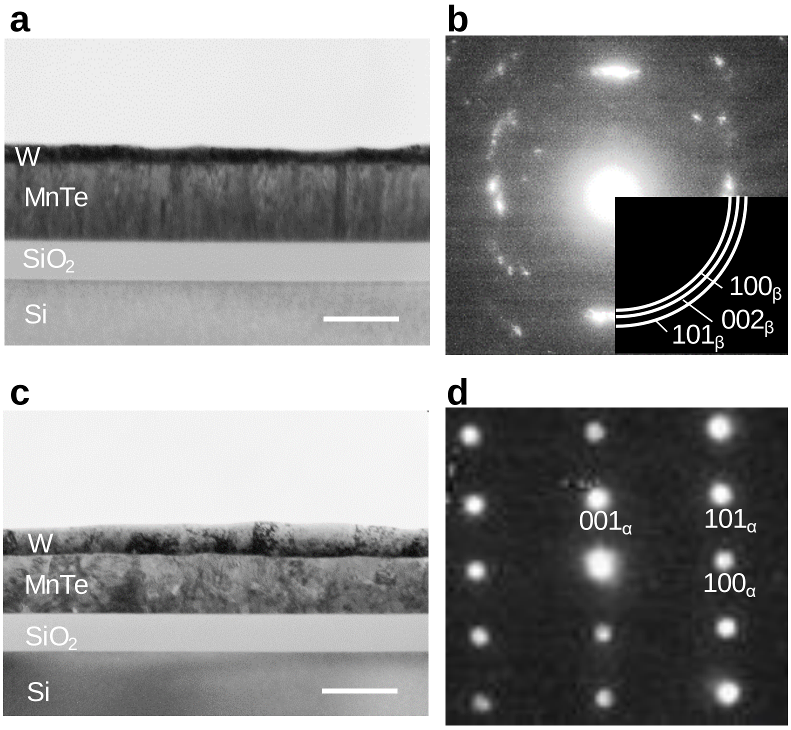

Supplementary Figure 2. Microstructure of MnTe films. (**a**) Transmission electron microscopy (TEM) bright-field image of the as-deposited MnTe film. Scale bar: 200 nm. (**b**) Electron diffraction pattern from the MnTe layer in (**a**). (**c**) TEM bright-field image of the annealed MnTe film at 500 °C. Scale bar: 200 nm. (**d**) Electron diffraction pattern from the MnTe layer in (**c**). The top W layer was deposited to prevent the surface oxidation of the MnTe film during annealing.

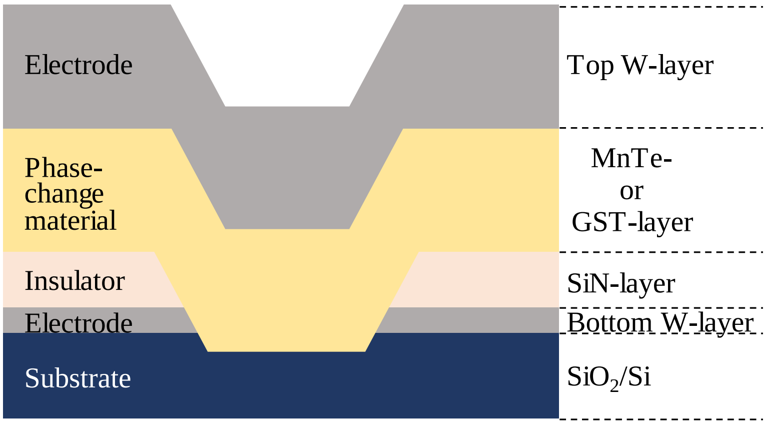

Supplementary Figure 3. Cross-sectional schematic view of the fabricated device.

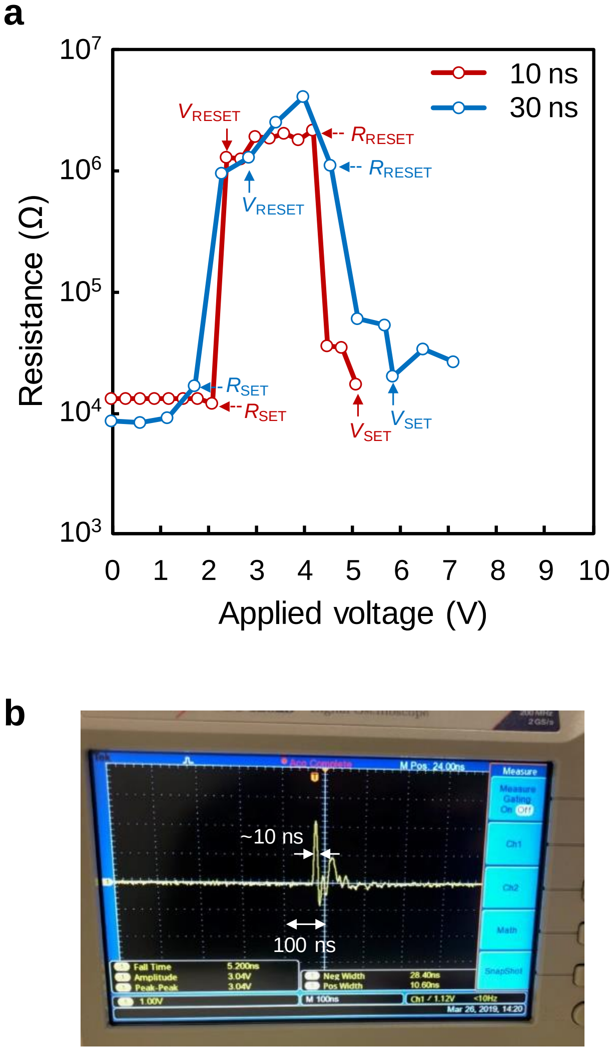

Supplementary Figure 4. Memory Operation in MnTe devices under short pulse width. (**a**) Resistance (R) versus voltage (V) curves of the MnTe devices obtained under 30 and 10 ns voltage pulse widths. The initial low-resistance state of the devices was obtained by annealing at 500 °C followed by cooling to room temperature (RT) without a holding time at 500 °C. The solid arrows indicate the critical voltages for the RESET (V_{RESET}) and the SET (V_{SET}) operations, and the dashed arrows indicate the resistance of the RESET (R_{RESET}) and SET (R_{SET}) states. These parameters were used in operation energy calculation. (**b**) Shape of the 10 ns voltage pulse obtained using an oscilloscope.

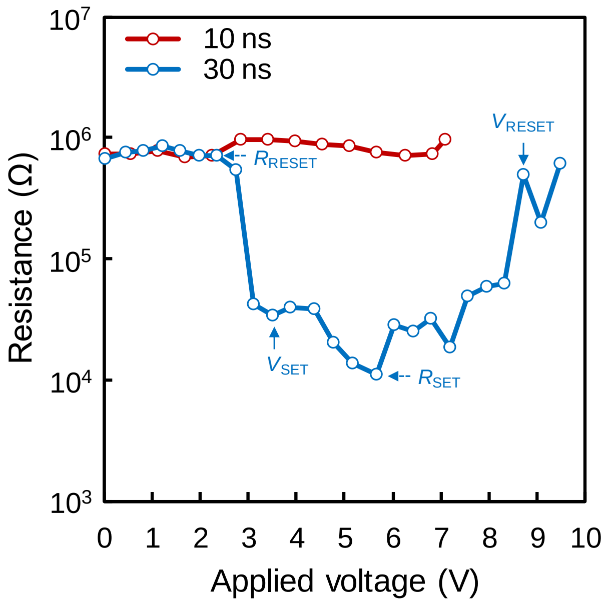

Supplementary Figure 5. Resistance (*R*) versus voltage (*V*) curves of $Ge_2Sb_2Te_5$ (GST225) devices. The fabricated GST225 devices were first annealed at 260 °C and then cooled to RT without a holding time at 260 °C. For the operation under 30 and 10 ns voltage pulses, the initial high-resistance state was obtained by applying a RESET voltage pulse of 22.6 V for 50 ns and 19.6 V for 50 ns. Under the 10 ns electrical pulse operation, the device did not work. The solid arrows indicate the critical voltages for the RESET (V_{RESET}) and the SET (V_{SET}) operations, and the dashed arrows indicate the resistance of the RESET (R_{RESET}) and the SET (R_{SET}) states. These parameters were used for the operation energy calculation.

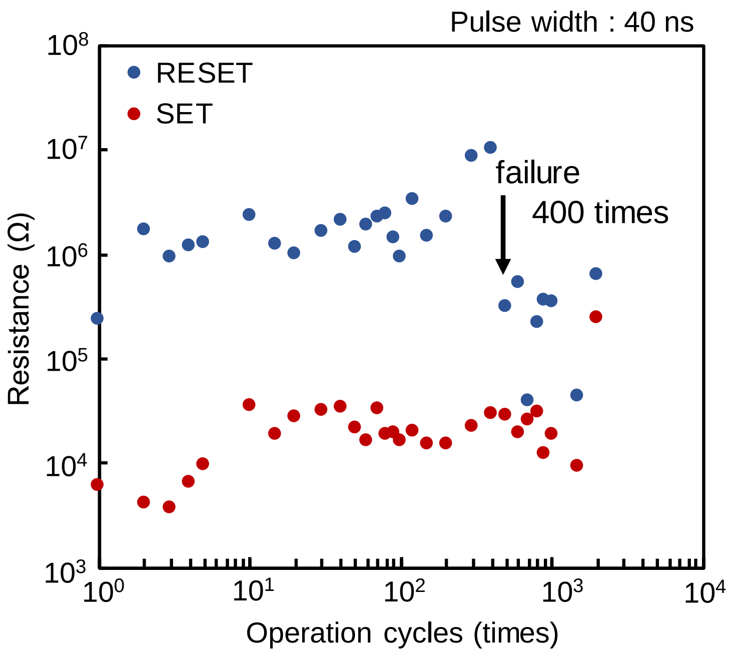

Supplementary Figure 6. Cyclic resistive switching behavior of the MnTe device. The blue and red circles indicate a high-resistance RESET state and a low-resistance SET state, respectively. In this experiment, the RESET and the SET pulse voltages were set to 1.7 V for 40 ns and 3.1 V for 40 ns. In this experiment, a 300 nm thick MnTe layer and a 500 nm thick top W layer were used.

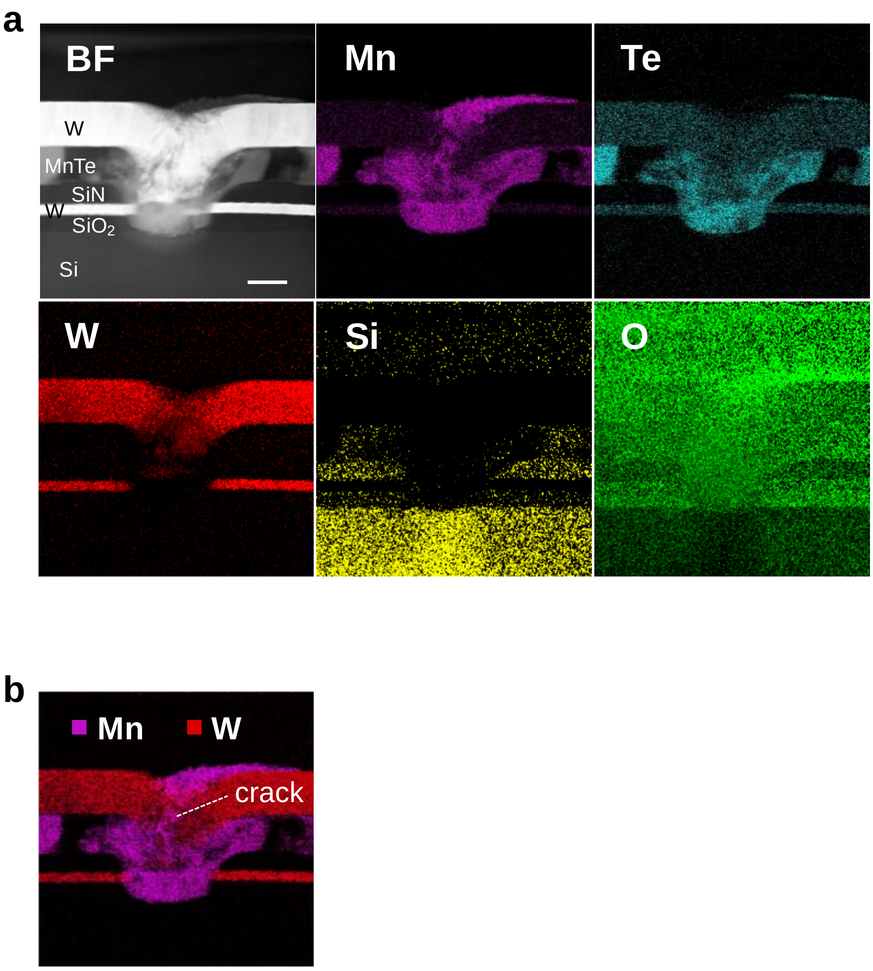

Supplementary Figure 7. Cross-sectional microstructure of the MnTe device after failure. (**a**) Cross-sectional bright-field scanning transmission electron microscopy (STEM) image and the corresponding STEM energy-dispersive X-ray spectrometry (STEM-EDS) images of Mn (purple), Te (light blue), W (red), Si (yellow), and O (green) of the MnTe device, which broke down after SET–RESET operation. Scale bar: 200 nm. We found that there are cracks in the top W electrode at the contact hole, since the upper W electrode has a concave shape. (**b**) Composite STEM-EDS image of Mn (purple) and W (red) indicating Mn diffused out to the surface through the crack in the W layer.

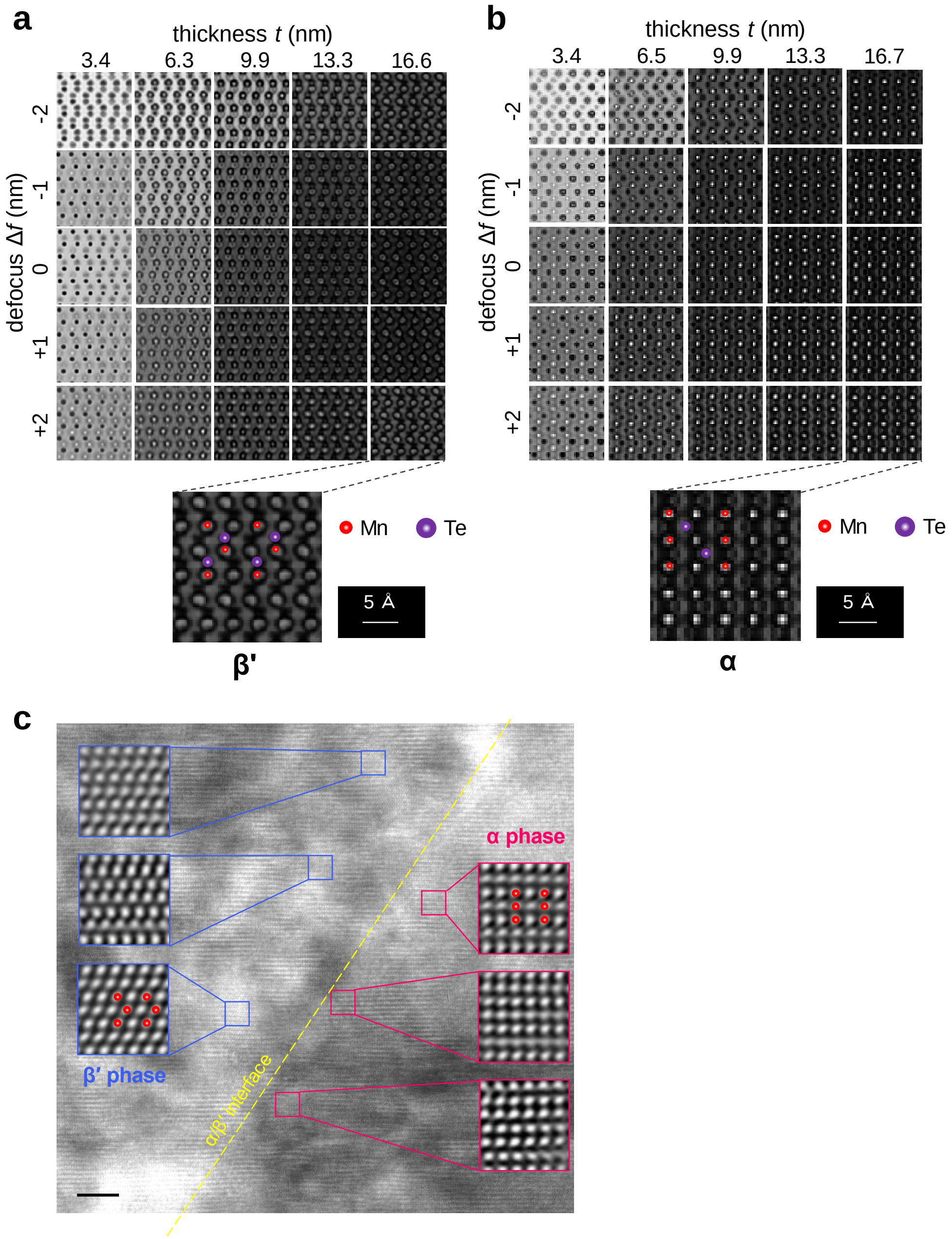

Supplementary Figure 8. High-resolution transmission electron microscopy (HR-TEM) image. Simulated thickness-defocus map of (**a**) the β′-phase and (**b**) the α-phase viewed from the [010] and [0$\bar{1}$0] directions, respectively, obtained by the multislice method for HR-TEM images. (**c**) HR-TEM image across the boundary between the active region and the matrix in the MnTe device. Scale bar: 5 nm. Inverse fast Fourier transform (IFFT) images from various areas: the blue lines are the active region, the β′-phase, and the red lines are the matrix, the α-phase. The best-fitted simulation results and the observed images are depicted at the bottom of (**a**) and (**b**). For the simulation, lattice parameters of a = 4.211 Å, c = 6.806 Å and a = 4.225 Å, c = 6.785 Å were used for the β′-phase and the α-phase, respectively. The IFFT images were obtained using Gatan DigitalMicrograph®.

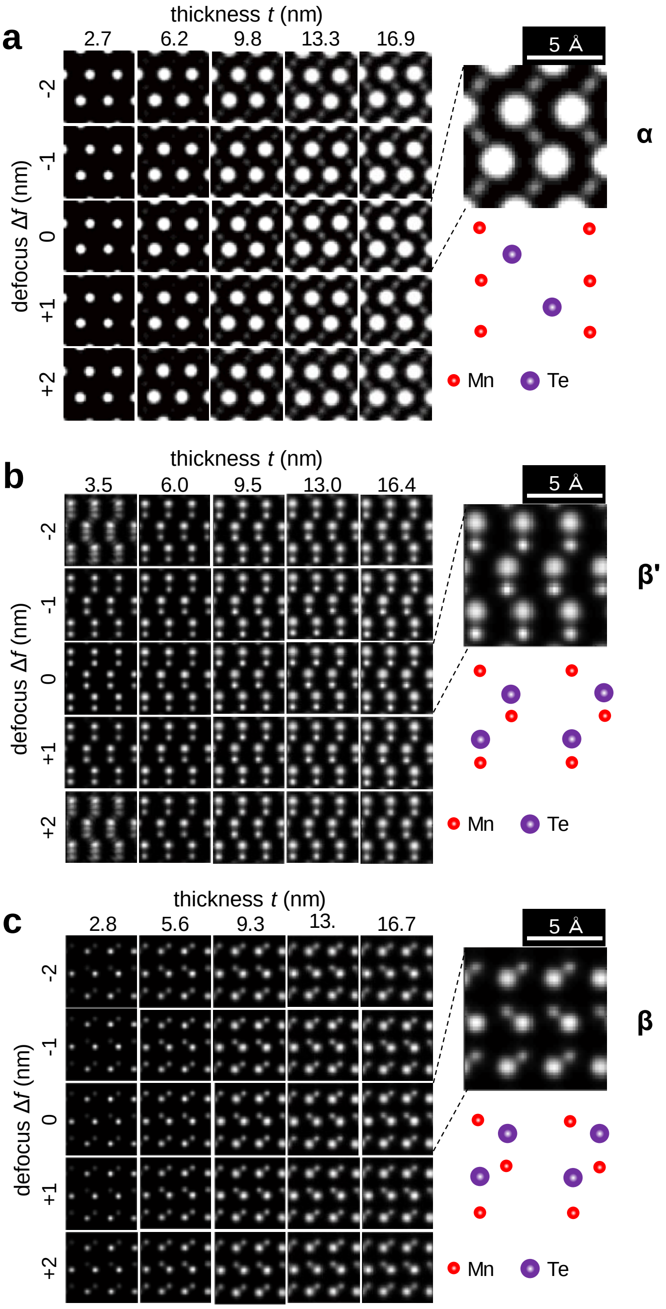

Supplementary Figure 9. Simulated Z-contrast images obtained by multislice method. Simulated thickness-defocus map of (**a**) the α-phase and (**b**) the β′-phase viewed from the $[0\bar{1}0]$ and [010] directions, respectively, obtained by the multislice method for the annular dark-field scanning transmission electron microscopy (ADF-STEM) images. The dark and bright spots indicate Mn and Te atoms, respectively. The best-fitted results with the observed images (Fig. 3b) are depicted on the right side of each map. The schematic atomic configurations of the α-phase and the β′-phase on the $(1\bar{2}0)$ and $(\bar{1}20)$ planes are also depicted, with the red and purple circles corresponding to Mn and Te atoms, respectively. (**c**) Simulated thickness-defocus map of the β-phase viewed from the [010] direction obtained by the multislice method for the ADF-STEM image, where the lattice parameters of a = 4.536 Å and c = 7.416 Å were used for the simulation.

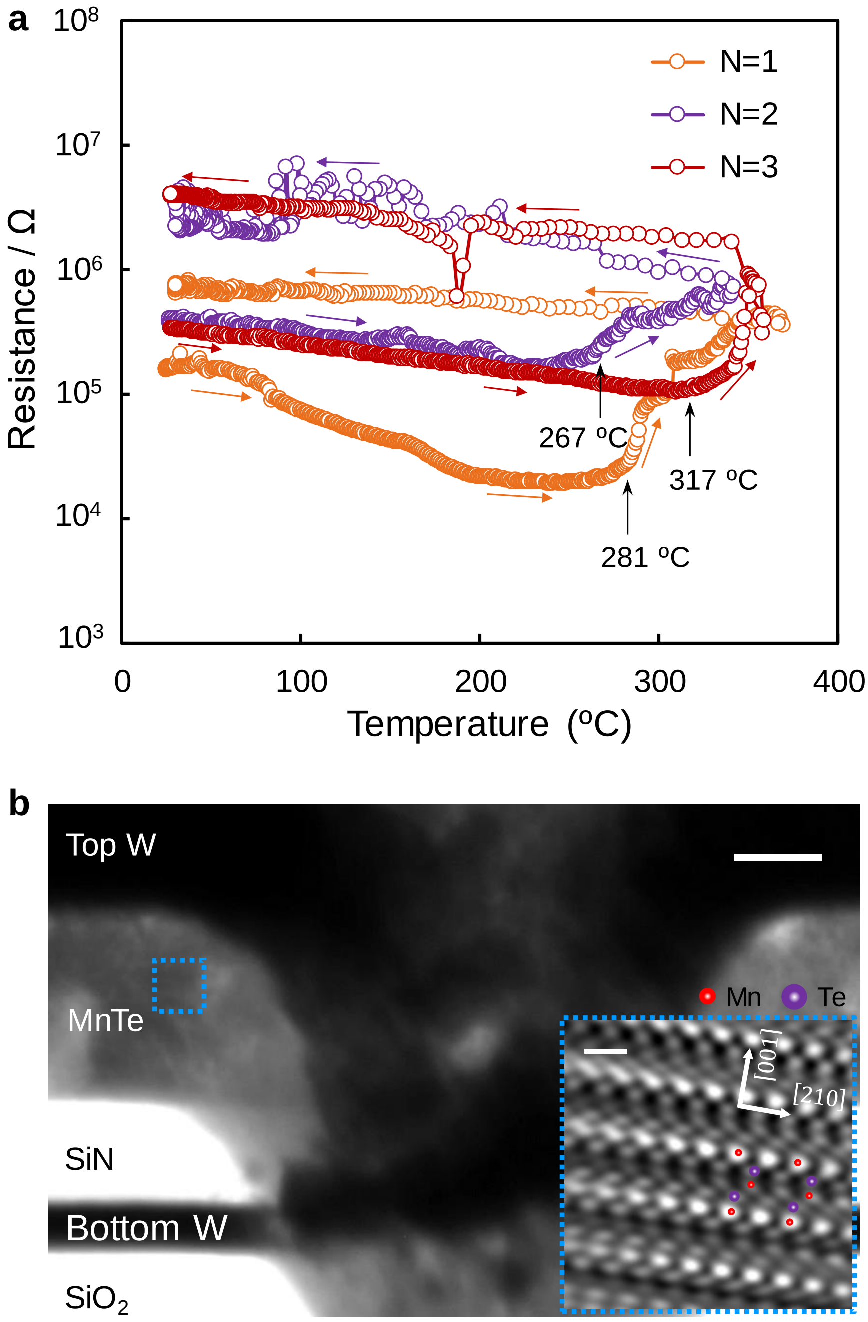

Supplementary Figure 10. Resistance change upon a transition from α to β′ in the MnTe device. (**a**) Temperature dependence of the resistance of the MnTe devices (N = 3). The initial state of the MnTe layer in the devices was an α-phase. All devices showed an increase in resistance at around 300°C, indicating a transition from the α-phase to the β′-phase. (**b**) Cross-sectional transmission electron microscopy (TEM) image of the MnTe device after heating up to 370°C. Scale bar: 100 nm. The inset shows an inverse fast Fourier transform (IFFT) image of the high-resolution TEM (HR-TEM) image taken from near the contact area enclosed by the blue dotted line. Scale bar: 5 Å.

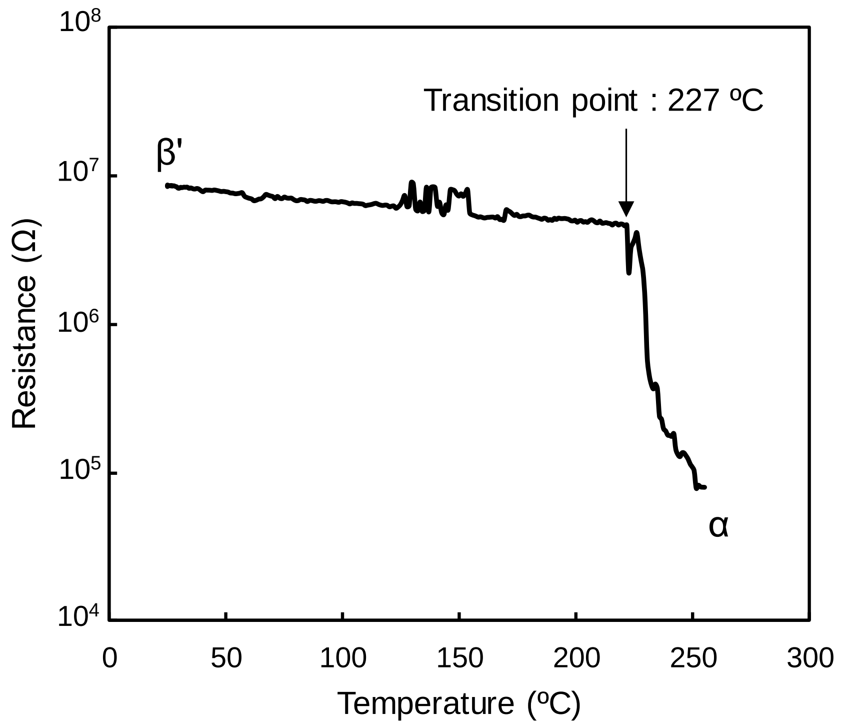

Supplementary Figure 11. Resistance change upon a transition from β′ to α in MnTe device. Temperature dependence of the resistance of the MnTe device after the RESET operation (5.7 V for 50 ns).

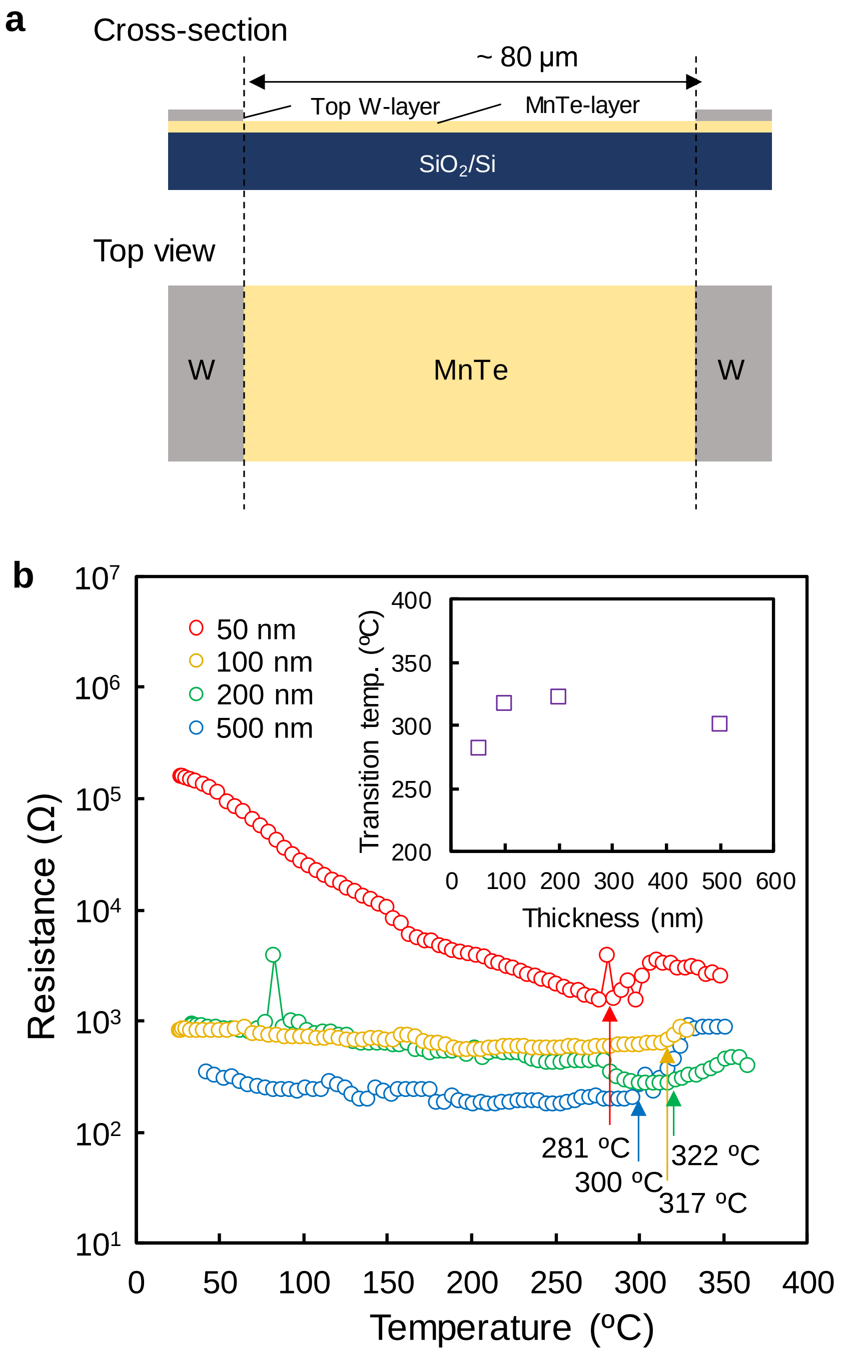

Supplementary Figure 12. Thickness dependence of the transition behavior from α to β′ in MnTe films. (**a**) Schematic description of the MnTe film for the investigation of the temperature dependence of the resistance (upper: cross-section, bottom: top view). (**b**) Temperature dependence of the resistance of the 50, 100, 150, and 200 nm thick α-MnTe films with patterned W electrodes on the surface. The inset shows the thickness dependence of the onset temperature (indicated by arrows), at which the resistance starts to increase, indicating the transition temperature from the α-phase to the β′-phase.

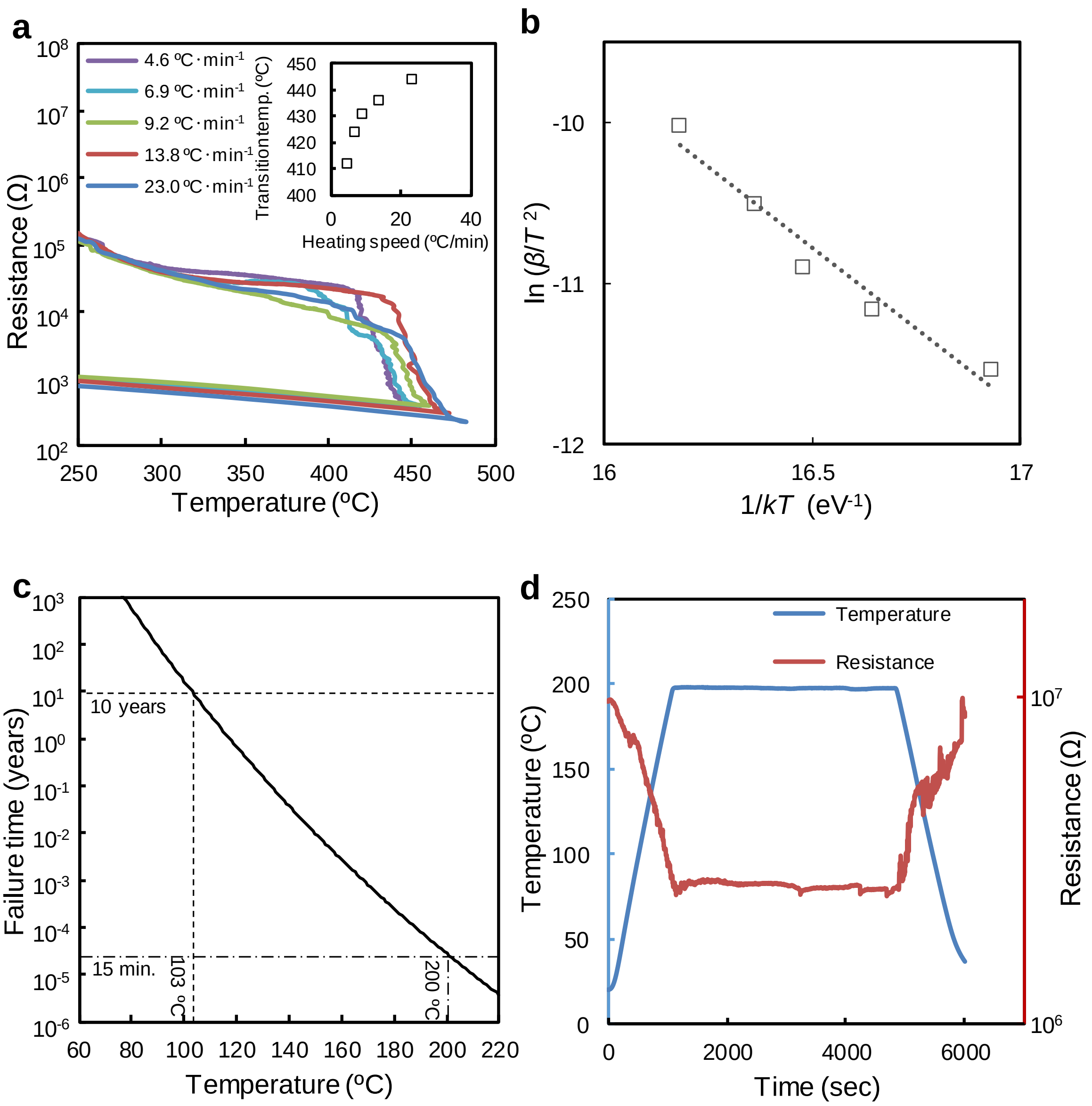

Supplementary Figure 13. Thermal stability of MnTe films and device. (**a**) Temperature dependence of the resistance of as-deposited MnTe films at various heating rates. (**b**) Kissinger plots for the activation energy of the β- to α-phase transition. From the slope of the plots, the activation energy was estimated to be 2.02 eV. (**c**) Plot of failure time vs. temperature for the MnTe film, which was obtained using the Ozawa method. Failure time was defined as the time when the transition started. (**d**) Resistance change as a function of annealing time at 200 °C in a RESET-operated MnTe device (RESET operation: 4.2 V for 50 ns).

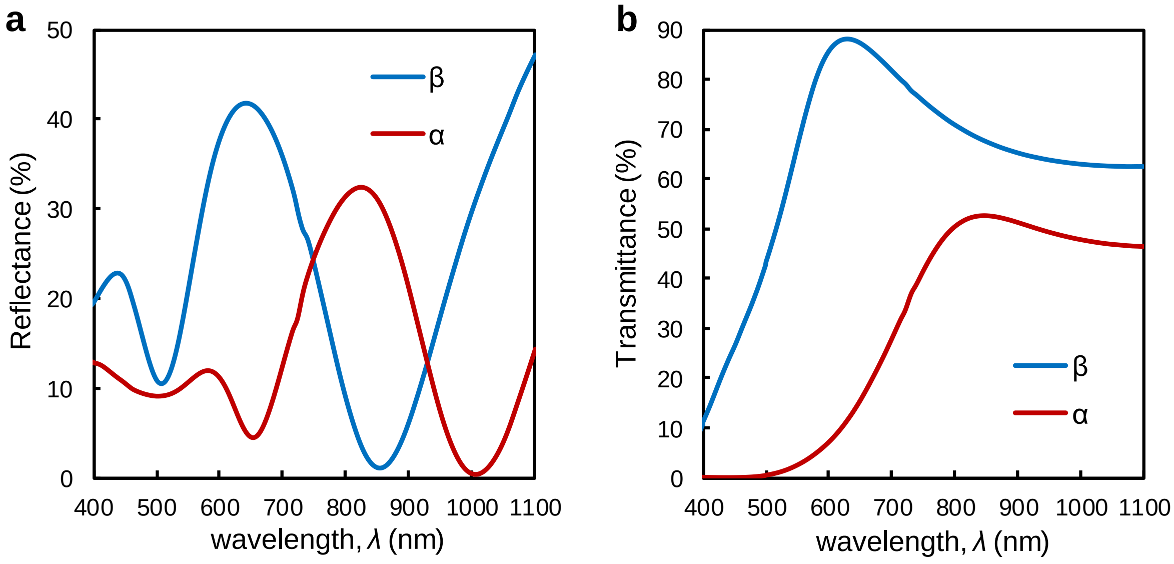

Supplementary Figure 14. Optical measurement in MnTe films using spectrophotometer. (**a**) Reflectance curves as a function of wavelength obtained in 150 nm thick MnTe films on a SiO_2/Si substrate. (**b**) Transmittance curves as a function of wavelength obtained in 100 nm thick MnTe films on a glass substrate.

Supplementary Table 1. Physical parameters in the α- and β-MnTe films. Optical bandgap (E_g), resistivity (ρ), carrier density (n), carrier mobility (μ), and conduction type in the α- and β-MnTe films were investigated.

	E_g (eV)	ρ (Ω·cm)	n (cm^{-3})	μ ($cm^2 \cdot V^{-1} \cdot S^{-1}$)	Conduction -type
α-MnTe	1.48	0.182	1.05×10^{19}	3.25	p
β-MnTe	2.50	9.37×10^{2}	6.78×10^{15}	0.982	p

Supplementary Table 2. Estimated operation energy in the MnTe and $Ge_2Sb_2Te_5$ (GST225) devices. Q_{RESET} and Q_{SET} are the RESET and the SET operation energies, respectively. Q_{total} is the total operation energy calculated from $Q_{\mathrm{RESET}} + Q_{\mathrm{SET}}$.

Material (pulse width)	Operation energy (pJ)		
	$\boldsymbol{Q_{total}}$	$\boldsymbol{Q_{SET}}$	$\boldsymbol{Q_{RESET}}$
MnTe (10 ns)	4.9	0.1	4.8
MnTe (30 ns)	15.6	1.0	14.6
MnTe (50 ns)	46.5	0.3	46.2
GST225 (10 ns)	-	-	-
GST225 (30 ns)	204.6	0.5	204.1
GST225 (50 ns)	795.1	0.7	794.4

Supplementary Note 1. X-ray diffraction measurement for the as-deposited MnTe film without a W capping layer

The X-ray diffraction (XRD) pattern in the as-deposited MnTe film (Supplementary Fig. 1) is fitted with the γ-phase with a sphalerite-type (zinc-blende) structure, since the Bragg reflection position of $(111)_\gamma$ is almost the same as that of $(002)_\beta$. The XRD pattern of the as-deposited MnTe film (1 μm thick) without a W capping layer in 2θ ranges from 20° to 55°, as shown in Supplementary Fig. 1. All Bragg reflection peaks are derived from those of a β-crystalline phase. This is strong evidence that the as-deposited MnTe film has a wurtzite β-phase, not a zinc-blende γ-phase.

Supplementary Note 2. Transmission electron microscopy microstructure of MnTe films

We observed the microstructures of the sputter-deposited MnTe film before and after annealing, as shown in Supplementary Fig. 2, where the as-deposited film was annealed at 500 °C, followed by cooling to room temperature (RT), with a holding time of 30 min at 500 °C. The transmission electron microscopy (TEM) results confirmed that the as-deposited film has a β-phase (wurtzite-type hexagonal [WZ-type] structure), with lattice parameters of a = 4.536 Å and c = 7.416 Å, whereas the 500 °C-annealed film has an α-phase with lattice parameters of a = 4.212 Å and c = 6.752 Å. These parameters are similar to the reported values in other previous studies.[1,2]

Supplementary Note 3. Resistive switching behavior of the MnTe and GST225 devices

The resistance (R) versus voltage (V) curves obtained under operation conditions, with voltage pulse widths of 30 and 10 ns for the MnTe device, are shown in Supplementary Fig. 4a. The MnTe device shows a resistive switching behavior at a 30 ns pulse width. A resistive switching operation with a 10 ns width (minimum width in the pulse generator used) was also attempted using an Agilent 33250A generator. An oscilloscope confirmed that a short voltage pulse with a half-width of 10 ns was obtained using this pulse generator, although there was a fluctuation in voltage after the application of the short pulse, as shown in Supplementary Fig. 4b. The MnTe device showed resistive switching even under the 10 ns electrical pulse. The fast operation speed of ~10 ns is the upper limit of our experimental setup. The intrinsic resistive switching speed of the MnTe device is believed to be even faster than this upper limit.

Supplementary Fig. 5 shows the R–V curves of the $Ge_2Sb_2Te_5$ (GST225) device obtained with a 30 and 10 ns voltage pulse width. The initial high-resistance state was obtained by applying a RESET voltage to the as-fabricated crystalline state. The GST225 device also showed resistive switching under the 30 ns electrical pulse, but not under the 10 ns pulse. These results indicate that the MnTe polymorphic-change material may have a faster operation speed compared to the GST225 phase-change material. Contrary to the GST225 device, V_{RESET} is lower than V_{SET} in the MnTe device, indicating that the RESET operation energy is very small.

Furthermore, we investigated the cyclic properties of the resistive switching behavior of the MnTe device. In this experiment, the RESET and SET operation conditions were fixed at 1.7 V for 40 ns and 3.1 V for 40 ns, respectively. The voltage pulse width was 40 ns in both cases. As shown in Supplementary Fig. 6, the results confirmed that the resistive switching of the MnTe device can be repeated more than 400 times.

Supplementary Note 4. Operation energy of the memory device

We calculated the total operation energy (Q_{total}) for the MnTe and the GST225 devices using the following equation:

$$Q_{\mathrm{total}} = Q_{\mathrm{RESET}} + Q_{\mathrm{SET}} = \left(V_{\mathrm{RESET}}{}^{2}/R_{\mathrm{SET}}\right) \times t + \left(V_{\mathrm{SET}}{}^{2}/R_{\mathrm{RESET}}\right) \times t \qquad (1)$$

where Q_{RESET} and Q_{SET} are the operation energies for the RESET and SET operations, respectively. V_{RESET} and V_{SET}, and R_{SET} and R_{RESET} are the RESET and SET voltages and device resistances, respectively, which are defined in Fig. 1b and Supplementary Fig. 4a and Fig. 5. The results under various pulse width conditions are shown in Supplementary Table 2. In both the MnTe and the GST225 devices, Q_{RESET} is much larger than Q_{SET}. It is worth noting that the Q_{RESET} value of the MnTe device is approximately more than one order of magnitude lower than that of the GST225 device. Consequently, the MnTe device shows about less than one-tenth of Q_{total}, as compared with the GST225 device. Such a low Q_{RESET} for the MnTe device implies melting-free phase transition from a low- to a high-resistance state.

Supplementary Note 5. Simulated high-resolution TEM and annular dark-field scanning TEM images analyzed by the multislice method

We simulated high-resolution TEM (HR-TEM) and dark-field scanning TEM (ADF-STEM) images from the [010] or [0$\bar{1}$0] direction in a hexagonal lattice using the multislice method in the QSTEM software.[3] The simulated thickness-defocus maps of the β′- and α-phases viewed from the [010] and [0$\bar{1}$0] direction for HR-TEM are shown in Supplementary Figs. 8a and 8b, respectively. In these figures, the accelerating voltage (V) the spherical aberration coefficient (C_{s}), and ΔE were fixed at 200 kV, 0 mm, and 0.5 eV, respectively. In the HR-TEM images, white spots indicate Mn atoms and black spots correspond to Te atoms.

Supplementary Fig. 8c shows a HR-TEM image of the area near the boundary between the α-matrix and the β′-phase, in the [0$\bar{1}$0] zone axis of the α-matrix. We obtained inverse fast Fourier transform (IFFT) images of the HR-TEM images from various areas. The left and right sides correspond to the β′-phase and α-matrix, respectively. The obtained images were well fitted to the simulation results at a film thickness of t = 16.6–16.7 nm and defocus Δf = +2 for both phases. The results indicate that there is a vague diagonal boundary from the bottom left to the top right.

The simulated thickness-defocus maps of the α- and β′-phases of the ADF-STEM images viewed from the [0$\bar{1}$0] and [010] direction are shown in Supplementary Figs. 9a and 9b, respectively. In these figures, the accelerating voltage (V), the spherical aberration coefficient (C_{s}), and ΔE were fixed at 200 kV, 0 mm, and 0.5 eV, respectively. In addition, ADF-STEM images of a β-MnTe phase were also simulated for comparison (Supplementary Fig. 9c). In the ADF-STEM images, the dark spots are Mn columns and the bright ones are Te columns. This is because their brightness depends on the atomic weight. The obtained images (Fig. 3b) were well fitted to the simulation results at a film thickness of t = 16.4–16.9 nm and defocus of Δf = 0 for both phases.

Supplementary Note 6. Transition temperature from the α-phase to the β′-phase

We investigated the transition behavior of the MnTe device upon annealing (heating) with a furnace chamber. We prepared three devices (N = 3) with a low-resistance state of an α-phase and then annealed them over 300 ºC. The results are shown in Supplementary Fig. 10a. We found that the devices show a resistance increase at around 300 ºC, indicating that a transition from the α-phase to the β′-phase occurs. Even when the devices were cooled down to RT, the high-resistance state was maintained. We observed the cross-sectional TEM microstructure of the device annealed up to 370 ºC, followed by air-cooling to RT. We confirmed that the β′-

phase exists near the contact area in the MnTe device, as shown in Supplementary Fig. 10b. These results strongly support the notion that a thermal-stress-induced displacive transformation occurs from the α-phase to the β′-phase, and the induced β′-phase can be quenched at RT. It has been noted that the resistance contrast obtained by heating the MnTe device is smaller than that obtained by local Joule heating, indicating that the local Joule heating generates a much larger compressive stress in the contact hole compared to the furnace heating. Therefore, the volume fraction of the β′-phase in the contact hole is considered to become large.

Supplementary Note 7. Estimation of an expected strain in the MnTe layer of the device

From the resistance change behavior of the MnTe device as a function of temperature, the transition temperature from the α-phase to the β′-phase was determined to be around 300 °C. Thus, we calculated the expected strain generated by the difference of thermal expansion between the MnTe layer and surrounding materials (i.e., the electrode and the insulator layer) upon heating at 300 °C. We used a thermal expansion value of 27.4 ppm·°C^{-1} (equivalent to [27.9 ppm·°C^{-1} for the a-axis + 26.8 ppm·°C^{-1} for the c-axis]/2) for MnTe and 4 ppm·°C^{-1} for the surrounding material (W and SiN). Here, for simplicity, only thermal expansion in the diameter direction of the contact hole is considered. The expected strain is estimated to be about 0.7%.

Supplementary Note 8. Transition temperature from the β′-phase to the α-phase

We evaluated the transition temperature from the displacive-transformation-induced β′-phase to the stable α-phase in the MnTe device using *in situ* two-point probe electrical resistance measurements. The results are shown in Supplementary Fig. 11. The high resistance of the β′-phase drops to a low value in the α-phase at about 227 °C, which is much lower than the transition temperature of the as-deposited β-phase to the stable α-phase (Fig. 1a). It is, however, still higher than that of conventional amorphous-to-crystalline phase-change materials (e.g., ~160 °C in GST). This indicates that the MnTe device has a better data retention ability.

Supplementary Note 9. Thickness dependence of transformation temperature

Supplementary Fig. 12 shows the temperature dependence of the resistance of 50, 100, 200, and 500 nm thick α-MnTe films with patterned W electrodes on the surface. The 100 nm thick MnTe film shows a slight increase in resistance at around 320 ºC, which is very similar to the MnTe device, as shown in Fig. 4. The resistance increase in the 100 nm thick film is considered to be caused by a transition from the α-phase to the β′-phase. The onset temperature at which the resistance starts to increase, indicating the transition temperature from the α-phase to the β′-phase, is plotted as a function of film thickness in the inset of Supplementary Fig. 12. The result indicates that the transformation temperature tends to decrease slightly in the thickness range of less than 100 nm with the decrease of film thickness. The results suggest that the memory performance depends on the device structure (e.g., memory layer thickness and contact size) because the MnTe film shows a thermal-stress-induced phase transformation.

It has been noted that the 50 nm thick film shows a larger negative temperature coefficient of resistance (TCR) than those of other thicknesses. It has been reported that the negative TCR in MnTe films increases with decreasing film thickness. Thanigaimani et al. demonstrated that MnTe films with a film thickness below 64 nm show a drastic increase in negative TCR with decreasing film thickness.[4] They suggested that MnTe films follow the Mayadas–Shatzkes model, which reflects the effect of electron scattering from phonons, film surface, and grain boundaries.

Supplementary Note 10. Thermal stability of the RESET-operated MnTe device

We evaluated the thermal stability of a RESET-operated MnTe device with the β′-phase. In this evaluation, we used the activation energy of the transition from the metastable β-phase to the stable α-phase to evaluate the thermal stability of the metastable β′-phase because of the structural similarity between the β-phase and the β′-phase. Supplementary Fig. 13a shows the temperature dependence of the as-deposited MnTe films at various heating rates. The inset shows the transition temperature as a function of the heating rate. The transition temperature increases with increasing heating rate. Supplementary Fig. 13b shows Kissinger plots for the activation energy of the transition of the β-phase to the α-phase in the MnTe film. The activation energy was estimated to be 2.02 eV. Based on the Ozawa method, using an activation energy of 2.02 eV and a transition temperature of 227 ºC, which was obtained in the MnTe device after the RESET operation, the MnTe memory layer is expected to show a 10-year lifetime at a maximum temperature of 103 ºC, as shown in Supplementary Fig. 13c. However, this value was estimated using the activation energy of the transition from the β-phase to the α-phase, and not the transition from the β′-phase to the α-phase. Therefore, we evaluated the thermal stability of the MnTe device with the RESET state at 200 ºC. Supplementary Fig. 13d shows the resistance change as a function of annealing time at 200 °C in a MnTe device after the RESET operation. The device resistance decreases and increases during the heating and cooling processes, respectively, owing to the semiconductor nature of the MnTe film, but the device does not show a clear change in resistance with phase transition during annealing at 200 ºC for 1 h. This indicates that the β′-phase shows a good thermal stability at 200 ºC for over 1 h. This result suggests that the thermal stability of the β′-phase is much better than the expected one, as shown in Supplementary Fig. 13c.

Supplementary Note 11. Reflectance and transmittance in a wide wavelength range

We could not measure the optical characteristics, such as the reflectance and transmittance as a function of wavelength, of the β′-phase because we could not obtain a large area of the β′-phase for the optical measurement. Therefore, we measured the optical spectra of the α-phase and the β-phase which has a fourfold-coordinated structure in the wavelength range between 400 and 1,100 nm. The results are shown in Supplementary Fig. 14.

Supplementary References

1 Mimasaka, M., Sakamoto, I., Murata, K., Fujii, Y. & Onodera, A. Pressure-induced phase transitions of MnTe. *J. Phys. C: Solid State Phys.* ***20***, 4689-4694 (1987).

2 Mori, S., Sutou, Y., Ando, D. & Koike, J. Optical and electrical properties of α-MnTe thin films deposited using RF magnetron sputtering. *Mater. Trans.* ***59***, 1506-1512 (2018).

3 Koch, C. Determination of core structure periodicity and point defect density along dislocations, PhD. thesis, 2002.

4 Thanigaimani, V. & Angadi M. A. Thickness dependence of temperature coefficient of resistance and neel temperature in MnTe films. *J. Mater. Sci. Lett.* ***12***, 1052-1056 (1993).
